# Urinary phthalate metabolites in relation to maternal serum thyroid and sex hormone levels during pregnancy: a longitudinal analysis

**DOI:** 10.1186/1477-7827-13-4

**Published:** 2015-01-17

**Authors:** Lauren E Johns, Kelly K Ferguson, Offie P Soldin, David E Cantonwine, Luis O Rivera-González, Liza V Anzalota Del Toro, Antonia M Calafat, Xiaoyun Ye, Akram N Alshawabkeh, José F Cordero, John D Meeker

**Affiliations:** Department of Environmental Health Sciences, University of Michigan School of Public Health, Ann Arbor, MI USA; Department of Medicine, Georgetown University, Washington, DC USA; Department of Obstetrics and Gynecology, Brigham and Women’s Hospital, Boston, MA USA; University of Puerto Rico Graduate School of Public Health, UPR Medical Sciences Campus, San Juan, Puerto Rico; Centers for Disease and Control and Prevention, Atlanta, GA USA; College of Engineering, Northeastern University, Boston, MA USA

**Keywords:** Biomarkers, Endocrine disruption, Epidemiology, Thyroid, Hormones, Urinary metabolites, Pregnancy, Phthalates

## Abstract

**Background:**

Increasing scientific evidence suggests that exposure to phthalates during pregnancy may be associated with an elevated risk of adverse reproductive outcomes such as preterm birth. Maternal endocrine disruption across pregnancy may be one pathway mediating some of these relationships. We investigated whether urinary phthalate metabolites were associated with maternal serum thyroid (free thyroxine [FT4], free triiodothyronine [FT3], and thyroid-stimulating hormone [TSH]), and sex (estradiol, progesterone, and sex hormone-binding globulin [SHBG]) hormone levels at multiple time points during pregnancy.

**Methods:**

Preliminary data (n = 106) were obtained from an ongoing prospective birth cohort in Northern Puerto Rico. We collected urine and serum sample at the first and third study visits that occurred at 18 +/- 2 and 26 +/- 2 weeks of gestation, respectively. To explore the longitudinal relationships between urinary phthalate metabolites and serum thyroid and sex hormone concentrations, we used linear mixed models (LMMs) adjusted for prepregnancy body mass index (BMI) and maternal age. An interaction term was added to each LMM to test whether the effect of urinary phthalate metabolites on serum thyroid and sex hormone levels varied by study visit. In cross-sectional analyses, we stratified BMI- and age-adjusted linear regression models by study visit.

**Results:**

In adjusted LMMs, we observed significant inverse associations between mono-3-carboxypropyl phthalate (MCPP) and FT3 and between mono-ethyl phthalate (MEP) and progesterone. In cross-sectional analyses by study visit, we detected stronger and statistically significant inverse associations at the third study visit between FT3 and MCPP as well as mono-carboxyisooctyl phthalate (MCOP); also at the third study visit, significant inverse associations were observed between FT4 and metabolites of di-(2-ethylhexyl) phthalate (DEHP). The inverse association between MEP and progesterone was consistent across study visits.

**Conclusions:**

In this group of pregnant women, urinary phthalate metabolites may be associated with altered maternal serum thyroid and sex hormone levels, and the magnitude of these effects may depend on the timing of exposure during gestation.

**Electronic supplementary material:**

The online version of this article (doi:10.1186/1477-7827-13-4) contains supplementary material, which is available to authorized users.

## Background

Phthalate diesters are commonly used as plasticizers in industrial applications [1]. Additionally, many household and consumer products, including flooring and wall coverings, food packaging, and cosmetics such as lotions and fragrances, contain phthalates [2–4]. Due to their ubiquitous use, human exposure to phthalates is widespread [5]. Because phthalates are not chemically bound to the plastics and other products that contain them, they can easily leach into household dust, food and water sources, and ambient air [1, 6, 7]. Consequently, human exposure to phthalates can occur through ingestion, inhalation, or dermal absorption [1, 3].

Growing evidence suggests that urinary concentrations of phthalate metabolites during pregnancy are associated with adverse reproductive outcomes including preterm birth and pregnancy loss [8, 9]. Hormonal production and regulation are critical for pregnancy maintenance and fetal growth and neurodevelopment; maternal endocrine disruption during pregnancy may be one pathway mediating some of these relationships [10–13].

Limited human health studies have examined the potential thyroid-altering effects of phthalates. In a cross-sectional study of men recruited from a U.S. fertility clinic, the urinary concentration of mono-(2-ethylhexyl) phthalate (MEHP), a metabolite of di-(2-ethylhexyl) phthalate (DEHP), was inversely associated with free thyroxine (3, 3’, 5, 5’-tetraiodo-L-thyronine, FT4) and total triiodothyronine (3, 3’, 5-triiodo-L-thyronine, T3) [14]. Urinary concentrations of DEHP metabolites were also inversely associated with total T3 and total and free T4, and positively associated with thyroid-stimulating hormone (thyrotropin, TSH) in a representative sample of U.S. adults (non-pregnant women and men) participating in the National Health and Nutritional Examination Survey (NHANES) [15]. Similar inverse relationships between urinary DEHP metabolites and total and free T3 (FT3) were reported in a cross-sectional study of Danish children [16]. While pervasive exposure to phthalates has been documented among pregnant women worldwide [17–22], human health studies investigating the effects of phthalate exposure during pregnancy on maternal thyroid function are scarce. Huang and colleagues [23] reported an inverse association between urinary concentrations of monobutyl phthalate (MBP), the metabolite of dibutyl phthalate (DBP), and both free and total T4 in the second trimester of Taiwanese pregnant women.

Animal and *in vitro* studies have also shown that phthalates can interfere with sex hormone concentrations, signaling, and/or function [24, 25], which may profoundly affect implantation, fetal development, and parturition [10, 26]. However, human data pertaining to the relationships between phthalates and sex hormones are limited. In men recruited through a U.S. fertility clinic, urinary MEHP was inversely associated with serum estradiol levels [27]. A positive association between urinary MEHP and sex hormone-binding globulin (SHBG) was also reported in separate cohort of fertile U.S. men [28]. In a recent study conducted among pregnant women in the U.S., an inverse association was found between urinary DEHP metabolite concentrations and serum testosterone concentrations, while no statistically significant relationship was observed for estradiol [29]. Additionally, Hart and coauthors [30] reported significantly inverse correlations between serum MEHP and SHBG concentrations at both 18 weeks and 36 weeks of gestation among pregnant women in Australia.

We are aware of no published investigations that longitudinally evaluate the potential thyroid-disrupting effects of environmental phthalate exposure among pregnant women. Furthermore, whether alterations in thyroid and sex hormone levels vary by time point of exposure during gestation remains largely undetermined and may have important influences on downstream hormone-mediated reproductive outcomes. In this preliminary analysis, we investigated the relationship between urinary phthalate metabolites and maternal serum thyroid (FT3, FT4, and TSH) and sex (estradiol, progesterone, and SHBG) hormone levels measured in samples collected at two time points in pregnancy from women participating in a prospective birth cohort in Puerto Rico.

## Methods

### Study population

The Puerto Rico Testsite for Exploring Contamination Threats (PROTECT) project is an ongoing prospective birth cohort in the Northern Karst Region of Puerto Rico designed to investigate the relationship between phthalates and other environmental contaminants and adverse pregnancy outcomes such as preterm birth. A previous study within this project reported greater concentrations of some urinary phthalate metabolites measured in Puerto Rican participants compared to women of reproductive age in the contiguous U.S. [31]. The current analysis included data collected from the first 106 pregnant women participating in the PROTECT project with urinary phthalate metabolites and serum thyroid and sex hormones measures completed as of November 2012. Study participants, aged 18 to 40 years, were recruited around 14 ± 2 weeks gestation from 7 prenatal clinics and hospitals throughout Northern Puerto Rico from 2010 to 2012. Participant recruitment and eligibility criteria as well as sample collection and processing are described in detail elsewhere [31]. Demographic information was obtained from questionnaires administered at the initial study visit. Spot urine samples were collected from each participant at three separate study visits (visit 1: 18 ± 2 weeks, visit 2: 22 ± 2 weeks, visit 3: 26 ± 2 weeks of gestation). Only urine samples from visits 1 and 3 were utilized in the present analyses because blood samples were not collected at visit 2. All participants in the present analysis provided urine and serum samples for at least one visit (visits 1 and/or 3). Upon collection and processing (e.g., aliquoting, centrifugation, and separation of blood into plasma and serum components), all urine and blood samples were frozen at -80°C until shipped overnight on dry ice to the analytical laboratories where samples were again stored at -80°C until analysis.

The study protocols were approved by the ethics and research committees of the participating institutions. The involvement of the Centers for Disease Control and Prevention (CDC) laboratory was determined not to constitute engagement in human subjects research. The study was described in detail to all participating women and all study participants gave informed consent.

### Measurement of phthalate metabolites

Available urine samples (N = 196 samples, N = 106 participants) were analyzed by the Centers for Disease Control and Prevention (CDC) laboratories, using protocols developed for NHANES, to measure urinary concentrations (free plus glucuronidated) of 11 phthalate metabolites: MEHP, MBP, mono-2-ethyl-5-hydroxyhexyl phthalate (MEHHP), mono-2-ethyl-5-oxohexyl phthalate (MEOHP), mono-2-ethyl-5-carboxypentyl phthalate (MECPP), mono-3-carboxypropyl phthalate (MCPP), mono-carboxyisooctyl phthalate (MCOP), mono-carboxyisononyl phthalate (MCNP), mono-benzyl phthalate (MBzP), mono-iso-butyl phthalate (MiBP), and mono-ethyl phthalate (MEP). The analytical method involved enzymatic deconjugation of the metabolites from their glucuronidated form, solid-phase extraction, separation by high-performance liquid chromatography, and detection by isotope-dilution tandem mass spectrometry [32]. The limits of detection (LOD) were in the low nanogram per milliliter range [31]. Concentrations below the LOD were assigned a value of LOD divided by the square root of 2 [33]. To account for urine dilution, urinary specific gravity (SG) was measured using a digital handheld refractometer (Atago Co., Ltd., Tokyo, Japan). For descriptive analyses, metabolite concentrations were standardized using the following formula: P_SG_ = P[(1.019 - 1)/(SG - 1)], where P_SG_ is the specific gravity-adjusted phthalate metabolite concentration (ng/mL), P is the observed phthalate metabolite concentration, 1.019 was the specific gravity population median, and SG is the specific gravity of the urine sample.

### Measurement of thyroid hormones

For 106 subjects, serum samples from visits 1 (N = 106) and 3 (N = 89) were available for measurement of FT3, FT4, and TSH at the Bioanalytical Core Laboratory of Georgetown University (Washington, DC). Limitations in sample volume contributed to differences in the number of samples per study visit available for analysis. Following an ultrafiltration step to separate the free hormones, FT3 and FT4 were measured using isotope dilution liquid chromatography tandem mass spectrometry per the methods described in detail previously [34–37]. TSH concentrations were measured using a solid-phase immunochemiluminometric assay (DPC Immulite, Diagnostic Products Corporation) according to the manufacturer’s instructions.

### Measurement of sex hormones

For 104 subjects, serum samples were analyzed for estradiol (visit 1: N = 103 samples; visit 3: N = 86 samples) and progesterone (visit 1: N = 104; visit 3: N = 89) using a chemiluminescence immunoassay (DPC Immulite, Diagnostic Products Corporation). Serum levels of SHBG from samples collected from 99 subjects (visit 1: N = 88; visit 3: N = 69) were determined using the same procedures. Similar to the thyroid hormone analyses, limitations in sample volume contributed to differences in available samples for each sex hormone/study visit. These analyses were also performed by the Bioanalytical Core Laboratory at Georgetown University (Washington, DC).

### Statistical analysis

Serum concentrations of FT4, FT3, estradiol, and SHBG closely approximated normality and were untransformed in statistical analyses. TSH, progesterone, and urinary phthalate metabolite concentrations were positively skewed and were logarithmically transformed prior to analyses. Because MEHP, MEOHP, MEHHP, and MECPP share a single parent compound, the sum of concentrations of the DEHP metabolites (ΣDEHP) was calculated from the molar sum (nmol/mL) of these four metabolites and log-transformed in statistical analyses. Means and standard deviations as well as selected percentiles were used to examine the distributions of the urinary phthalate metabolites, thyroid hormones, and sex hormones. Geometric means and standard deviations were calculated for non-normally distributed variables.

Pearson correlations were calculated to assess the relationships between continuous variables. To test the differences in the mean concentrations of sex and thyroid hormones by visit, we used linear mixed models (LMMs) with serum hormones levels regressed on study visit and included a random intercept for subject ID to account for intra-individual correlation of repeated measurements over time. Urinary phthalate metabolites and sex and thyroid hormones were tested for associations with demographic variables to examine potential confounding. We used LMMs with one phthalate metabolite concentration measure (as exposure) and one outcome per model to explore the longitudinal relationships between urinary phthalate metabolites and sex and thyroid hormone concentrations. Crude models were adjusted for urinary specific gravity and study visit. Full models additionally included maternal prepregnancy body mass index (BMI) and age, both measured at the initial study visit. BMI was modeled as a categorical variable (≤25 kg/m^2^, >25 and ≤30 kg/m^2^, >30 kg/m^2^) and age was modeled as a continuous variable. BMI and age were included as covariates because of their potential influence on urinary phthalate metabolite concentrations [38] and sex and thyroid hormone levels [39–41]. A likelihood ratio test for fixed effects was used to identify additional potential covariates in full models. An interaction term was added to each full LMM to test whether the effect of urinary phthalate metabolites on thyroid and sex hormone serum levels varied by study visit.

In a secondary analysis, we used linear regression models with one phthalate metabolite concentration and one outcome per model to investigate the cross-sectional relationships between urinary phthalate metabolites and sex and thyroid hormone serum concentrations at each study visit (visits 1 and 3). These models were also adjusted for maternal BMI, age, and urinary specific gravity. To enhance interpretability, all regression coefficients and associated 95% confidence intervals (CIs) generated from the LMMs and linear regression models were expressed as the percent change in hormone serum levels for an interquartile range (IQR) increase in urinary phthalate metabolite concentrations. Associations were considered statistically significant at the 5% level and marginally significant at the 10% level. All statistical analyses were conducted using SAS version 9.2 (SAS Institute Inc., Cary, NC).

## Results

Table 1 shows the distribution of sociodemographic characteristics of the 106 pregnant women included in the present analysis. The study participants were generally highly educated (79.2% had at least a college education), and did not smoke during pregnancy (94.3%). Approximately half (55.7%) of the women had a prepregnancy BMI ≤25 kg/m^2^. No statistically significant differences were observed between the demographic characteristics of study participants with measurable thyroid and sex hormone concentrations at visit 1 vs. those with measurable thyroid and sex hormone concentrations at visit 3 (chi-square test p-values > 0.05). Based on the eligibility criteria, no participants used oral contraceptives within three months prior to pregnancy or underwent *in vitro* fertilization as a method of assisted reproductive technology, and all participants were free of known medical or obstetric complications.

**Table 1:** **Sample characteristics of 106 pregnant women participating in PROTECT project (2010–2012)**

Variable	Mean ± SD or n (%)
Maternal age at enrollment (years)	27.1 ± 4.8
Maternal education	
Missing	3 (2.8)
<High school	12 (11.3)
High school/equivalent	7 (6.6)
College	84 (79.2)
Annual household income (US$)	
Missing	17 (16.0)
$20,000	45 (42.4)
≥$20,000 to < $50,000	27 (25.5)
≥$50,000	17 (16.0)
Race	
Missing	3 (2.8)
White	52 (49.1)
Mixed	39 (36.8)
Other	12 (11.3)
Prepregnancy BMI (kg m^-2^)	
Missing	4 (3.8)
≤25	59 (55.7)
>25 to ≤30	31 (29.2)
>30	12 (11.3)
Smoked during pregnancy	
Missing	5 (4.7)
Yes	1 (0.9)
No	100 (94.3)
Alcohol use at first visit during pregnancy	
Missing	4 (3.8)
Yes	12 (11.3)
No	90 (84.9)

PROTECT, Puerto Rico Testsite for Exploring Contamination Threats, SD, standard deviation, BMI, body mass index.

The distributions of the 11 urinary phthalate metabolite concentrations across pregnancy have been previously investigated in this cohort [31]. Greater than 90% of the measured concentrations of all 11 urinary phthalate metabolites were detectable [31]. Concentrations for a majority of the metabolites measured were greater in the Puerto Rican participants when compared to women of reproductive age in the contiguous U.S. [31]. For example, the geometric mean concentration of urinary MEHP (unadjusted for urinary dilution) was more than twice as high in the Puerto Rican cohort than the corresponding concentration found in women of reproductive age participating in the 2009–2010 NHANES study (3.3 vs. 1.6 ng/mL, respectively) [31]. Spearman correlations between the majority of urinary metabolites were modest (R = 0.23-0.45), while strong correlations were observed between all urinary metabolites of DEHP (MEHP, MEHHP, MEOHP, and MECPP; R > 0.83) [31]. With the exception of MCOP, no statistically significant differences were observed in the geometric mean concentrations between study visits for any of the other urinary phthalate metabolites (Additional file 1: Table S1).

The distributions of the thyroid and sex hormones are presented in Table 2. All of the measured concentrations of the hormones were detectable. We found a significantly positive Pearson correlation between estradiol and progesterone (R = 0.66, p < 0.001). Weak but significant positive correlations were observed between FT4 and FT3 (R = 0.17, p = 0.02), and between SHBG and both progesterone (R = 0.23, p = 0.004) and estradiol (R = 0.29, p = 0.0002). Age was inversely correlated with thyroid and sex hormone levels (Additional file 2: Table S2). With the exception of TSH, LMMs showed that thyroid hormone levels differed significantly by study visit (Table 3). We found significantly higher mean levels of serum FT3 and FT4 at visit 1 compared to corresponding levels at visit 3. Conversely, the mean serum levels of all three sex hormones were significantly lower at visit 1 compared to visit 3.

**Table 2:** **Distributions of serum hormone concentrations (N = 106 pregnant women)**

Biomarker	N	Mean (SD)	Selected percentiles				
			25th	50th	75th	95th	Max.
**Thyroid hormones**							
TSH (uIU/mL)†	195	1.13 (1.62)	0.81	1.10	1.51	2.83	4.29
Free T3 (pg/mL)	195	3.94 (0.60)	3.50	3.90	4.40	4.90	5.70
Free T4 (ng/dL)	195	1.39 (0.32)	1.20	1.40	1.50	1.90	3.10
Sex hormones*							
Progesterone (ng/mL)†	193	62.8 (1.60)	45.7	57.3	82.0	140	374
Estradiol (ng/mL)	189	8.16 (4.06)	4.79	7.58	11.8	15.0	15.0
SHBG (nmol/L)	157	368 (109)	293	352	428	563	736

SD, standard deviation, TSH, thyroid-stimulating hormone, SHBG, sex hormone-binding globulin.

†Geometric mean and geometric standard deviation reported.

*Limitations in sample volume contributed to differences in available samples for each sex hormone.

**Table 3:** **Mean serum hormone concentrations (SD) by visit during gestation (N = 106 pregnant women)**

Biomarker	Visit 1 (14–20 weeks)	Visit 3 (24–28 weeks)	p-value*
**Thyroid Hormones**			
TSH (uIU/mL)†	1.10 (1.60)	1.17 (1.65)	0.25
Free T3 (pg/mL)	4.01 (0.61)	3.86 (0.59)	0.02
Free T4 (ng/dL)	1.45 (0.31)	1.32 (0.32)	0.001
**Sex Hormones**			
Progesterone (ng/mL)†	46.9 (1.37)	88.4 (1.48)	<0.001
Estradiol (ng/mL)	5.70 (2.48)	11.1 (3.60)	<0.001
SHBG (nmol/L)	349 (108)	391 (106)	0.002

SD, standard deviation, TSH, thyroid-stimulating hormone, SHBG, sex hormone-binding globulin.

†Geometric mean and geometric standard deviation reported.

*Test for fixed effects from linear mixed models with random intercepts.

Table 4 shows the fully adjusted longitudinal associations between urinary phthalate metabolite concentrations and serum hormones concentrations from LMMs. Each model contained random intercepts only for subject ID to account for intra-individual correlation, as addition of random slopes did not improve the model fit. A likelihood ratio test for fixed effects indicated that maternal education did not have a significant effect on serum hormone levels. Thus, prepregnancy BMI, maternal age, study visit, and urinary specific gravity were the only covariates retained in the final models. The crude regression results were similar to the adjusted results (not shown). We detected a significant inverse relationship between MCPP and FT3, where an IQR increase in MCPP was associated with a 2.89% decrease in FT3 (95% CI = -5.65 to -0.02, p = 0.049). We also observed a significant inverse association between MEP and progesterone. An IQR increase in MEP was associated with a 10.6% decrease in progesterone (95% CI = -17.6 to -2.84, p = 0.01). We detected no significant associations between serum hormones levels and ΣDEHP urinary metabolites in longitudinal analyses. Similarly, null results were observed for individual DEHP metabolites, with the exception of a marginally significant inverse association found between MEHHP and SHBG (percent change in outcome with IQR increase [%Δ] = -4.62, 95% CI = -10.0 to 0.75, p = 0.09). Most urinary phthalate metabolites were positively associated with TSH, although none of these relationships were statistically significant. We also found no statistically significant associations for FT4 or estradiol in the longitudinal analyses.

**Table 4:** **Longitudinal analysis: percent change (95% CIs) in thyroid and sex hormone concentrations in relation to interquartile range increase in urinary phthalate metabolite concentration**

Analyte	% Change (95% CI)	p-value	% Change (95% CI)	p-value	% Change (95% CI)	p-value
	Free T3 (n = 181 observations)		Free T4 (n = 181 observations)		ln-TSH (n = 181 observations)	
MBzP	0.18 (-3.42, 3.77)	0.92	0.58 (-4.45, 5.56)	0.82	5.51 (-4.40, 16.5)	0.28
MBP	1.33 (-1.90, 4.67)	0.42	0.63 (-4.00, 5.21)	0.79	3.28 (-5.58, 12.8)	0.48
MiBP	0.19 (-2.86, 3.14)	0.90	1.14 (-3.06, 5.42)	0.59	4.64 (-3.73, 13.0)	0.28
MEP	1.11 (-2.74, 4.96)	0.57	2.68 (-2.68, 7.83)	0.33	0.05 (-10.1, 11.3)	0.99
MCPP	-2.89 (-5.65, -0.02)	0.049*	-2.01 (-6.12, 2.10)	0.34	1.60 (-6.05, 9.88)	0.68
MCOP	-2.03 (-5.08, 0.98)	0.18	-1.11 (-5.26, 3.13)	0.61	-2.52 (-10.3, 6.13)	0.56
MCNP	0.48 (-2.03, 2.94)	0.71	-1.64 (-5.28, 1.93)	0.36	3.50 (-2.98, 10.4)	0.30
ΣDEHP	-0.28 (-3.63, 0.30)	0.87	-0.93 (-5.56, 3.79)	0.70	7.21 (-1.87, 16.6)	0.12
	**Estradiol (n = 175 observations)**		**SHBG (n = 147 observations)**		**ln-progesterone (n = 179 observations)**	
MBzP	0.86 (-8.52, 10.3)	0.86	4.89 (-1.52, 11.3)	0.13	3.16 (-4.56, 11.5)	0.67
MBP	2.65 (-5.90, 11.2)	0.54	-0.65 (-6.29, 4.98)	0.82	3.71 (-3.44, 11.2)	0.31
MiBP	1.56 (-6.27, 9.39)	0.69	1.19 (-4.25, 6.61)	0.66	2.48 (-3.96, 9.49)	0.46
MEP	1.60 (-8.49, 11.7)	0.75	-2.24 (-9.43, 4.97)	0.54	-10.6 (-17.6, -2.84)	0.01*
MCPP	-3.86 (-11.5, 3.80)	0.32	-1.27 (-6.40, 3.86)	0.62	-4.31 (-10.2, 1.98)	0.17
MCOP	-3.59 (-11.4, 4.24)	0.36	-0.93 (-6.44, 4.58)	0.74	-4.83 (-11.0, 1.57)	0.13
MCNP	-2.03 (-8.50, 4.41)	0.53	-0.66 (-4.85, 3.46)	0.75	-2.06 (-7.22, 3.28)	0.43
ΣDEHP	-0.56 (-9.17, 8.06)	0.90	-4.11 (-9.83, 1.62)	0.16	1.79 (-5.17, 9.39)	0.62

CI, confidence interval, TSH, thyroid-stimulating hormone, SHBG, sex hormone-binding globulin. Linear mixed models include a random intercept for subject ID and are adjusted for age at enrollment, prepregnancy body mass index, as well as urinary specific gravity and study visit.

*P < 0.05.

In our examination of the potential interaction between study visit and urinary phthalate metabolite concentrations in longitudinal analyses, we observed that study visit significantly modified the relationships between FT3 and MBzP (p = 0.03) and MCPP (p = 0.03), between FT4 and MiBP (p = 0.01) and ΣDEHP metabolites (p = 0.048), and between TSH and MBP (p = 0.04). We found no statistically significant interactions between study visit and urinary phthalate metabolites for any of the sex hormones examined (data not shown).

Table 5 presents the results from cross-sectional analyses by study visit for associations between urinary phthalate metabolites and serum thyroid hormone concentrations. At visit 1, we observed a statistically significant positive association between MiBP and FT4 (%Δ =4.95, 95% CI = 0.27 to 9.28, p = 0.04). MBzP and ΣDEHP metabolites were also positively, though not significantly, associated with FT4 at visit 1. At visit 3, urinary phthalate metabolites were generally inversely associated with FT4. At this visit, we found a statistically significant inverse association between ΣDEHP and FT4 (%Δ = -8.02, 95% CI = -15.3 to -0.80, p = 0.03). Urinary phthalate metabolites were generally positively associated with TSH at each study visit, and suggestive associations were observed with MBzP at visit 1 and MEP at visit 3. While FT3 was inversely associated with both MCPP and MCOP at visit 1, these effects were greater and statistically significant at visit 3.

**Table 5:** **Cross-sectional analysis: percent change (95% CIs) in thyroid hormone concentrations in relation to interquartile range increase in urinary phthalate metabolite concentration by visit during gestation**

Visit 1 (16–20 weeks)						
Analyte	% Change (95% CI)	p-value	% Change (95% CI)	p-value	% Change (95% CI)	p-value
	Free T3 (n = 100 observations)		Free T4 (n = 100 observations)		ln-TSH (n = 100 observations)	
MBzP	1.87 (-2.55, 6.37)	0.40	2.44 (-2.77, 7.65)	0.36	12.9 (-0.93, 28.2)	0.07†
MBP	-0.72 (-4.94, 3.29)	0.73	0.26 (-4.58, 5.04)	0.92	1.29 (-10.2, 13.7)	0.83
MiBP	-1.64 (-5.55, 2.42)	0.43	4.95 (0.27, 9.28)	0.04*	1.09 (-10.3, 13.9)	0.86
MEP	-0.20 (-5.37, 5.02)	0.94	-0.05 (-6.10, 6.10)	0.99	0.77 (-13.6, 17.7)	0.92
MCPP	-1.04 (-4.54, 2.62)	0.57	1.41 (-2.83, 5.80)	0.50	-2.57 (-12.7, 8.69)	0.64
MCOP	-0.50 (-4.11, 2.93)	0.78	-0.44 (-4.58, 3.76)	0.83	-0.39 (-10.3, 10.7)	0.94
MCNP	1.05 (-2.27, 4.49)	0.53	0.43 (-3.47, 4.37)	0.83	-2.68 (-11.9, 7.35)	0.58
ΣDEHP	-1.62 (-6.05, 2.85)	0.47	3.09 (-2.17, 8.27)	0.25	7.14 (-6.12, 22.3)	0.30
**Visit 3 (24–28 weeks)**						
	**Free T3 (n = 81 observations)**		**Free T4 (n = 81 observations)**		**ln-TSH (n = 81 observations)**	
MBzP	-2.67 (-8.3, 2.86)	0.34	-2.97 (-12.1, 6.07)	0.51	-4.62 (-20.4, 13.5)	0.58
MBP	-0.57 (-5.68, 4.34)	0.82	-1.46 (-9.67, 6.74)	0.72	9.57 (-6.27, 27.3)	0.25
MiBP	-2.51 (-6.81, 1.70)	0.24	-4.70 (-11.8, 2.08)	0.17	2.74 (-9.94, 17.9)	0.68
MEP	2.76 (-2.68, 8.44)	0.32	4.93 (-3.81, 13.7)	0.27	18.1 (0.00, 37.8)	0.05†
MCPP	-5.93 (-10.2, -1.75)	0.01*	-5.11 (-12.5, 2.22)	0.17	2.41 (-11.1, 17.7)	0.74
MCOP	-5.83 (-10.8, -0.58)	0.03*	-3.02 (-11.5, 5.38)	0.47	-3.35 (-17.1, 13.6)	0.67
MCNP	-1.44 (-5.92, 3.10)	0.54	-6.34 (-14.0, 0.99)	0.09†	5.39 (-8.50, 21.3)	0.46
ΣDEHP	0.05 (-4.49, 4.49)	0.98	-8.02 (-15.3, -0.80)	0.03*	2.79 (-10.8, 18.6)	0.70

CI, confidence interval, TSH, thyroid-stimulating hormone. Linear regression models adjusted for age at enrollment, prepregnancy body mass index, and urinary specific gravity.

*P < 0.05.

†P < 0.10.

The results of cross-sectional analyses for the relationships between urinary phthalate metabolites and sex hormone concentrations are presented in Table 6. We observed a marginally significant inverse relationship between MEP and SHBG at visit 1 (%Δ = -10.1, 95% CI = -21.8 to 1.65, p = 0.09), and a null association at visit 3. The inverse association between MEP and progesterone observed in the longitudinal analyses appeared consistent across study visits.

**Table 6:** **Cross-sectional analysis: percent change (95% CIs) in sex hormone concentrations in relation to interquartile range increase in urinary phthalate metabolite concentration by visit during gestation**

Visit 1 (16–20 weeks)						
Analyte	% Change (95% CI)	p-value	% Change (95% CI)	p-value	% Change (95% CI)	p-value
	Estradiol (n = 97 observations)		SHBG (n = 83 observations)		ln-progesterone (n = 98 observations)	
MBzP	-5.65 (-18.9, 7.57)	0.40	4.99 (-4.43, 14.4)	0.29	1.88 (-6.60, 11.3)	0.66
MBP	0.20 (-10.3, 14.2)	0.75	4.13 (-3.66, 11.9)	0.29	5.68 (-2.41, 13.7)	0.18
MiBP	-0.99 (-13.1, 11.1)	0.87	-0.92 (-9.77, 7.28)	0.77	5.56 (-2.46, 13.9)	0.18
MEP	0.03 (-15.5, 15.6)	1.00	-10.1 (-21.8, 1.65)	0.09†	-12.15 (-20.5, -2.99)	0.01*
MCPP	-2.93 (-14.4, 8.54)	0.61	-2.66 (-10.6, 5.27)	0.51	-0.42 (-7.61, 7.34)	0.91
MCOP	0.70 (-9.86, 11.3)	0.90	0.86 (-6.74, 8.48)	0.82	-0.57 (-7.27, 6.62)	0.87
MCNP	2.43 (-7.42, 12.3)	0.63	-0.65 (-7.75, 6.45)	0.86	-0.58 (-6.85, 6.11)	0.86
ΣDEHP	-7.50 (-20.9, 5.92)	0.27	-5.93 (-15.4, 3.54)	0.22	2.37 (-6.23, 11.9)	0.59
**Visit 3 (24–28 weeks)**						
	**Estradiol (n = 78 observations)**		**SHBG (n = 64 observations)**		**ln-progesterone (n = 81 observations)**	
MBzP	4.38 (-7.12, 15.9)	0.45	6.66 (-4.98, 18.3)	0.26	8.02 (-5.12, 23.4)	0.24
MBP	6.55 (-3.60, 16.7)	0.20	5.22 (-5.18, 15.7)	0.32	8.19 (-3.61, 21.0)	0.18
MiBP	2.42 (-5.96, 10.8)	0.57	3.46 (-4.89, 11.8)	0.41	2.86 (-6.81, 13.8)	0.58
MEP	1.36 (-9.57, 12.3)	0.81	1.00 (-10.4, 12.4)	0.86	-9.43 (-20.1, 2.51)	0.11
MCPP	-0.48 (-9.45, 8.48)	0.91	4.76 (-3.69, 13.2)	0.26	-3.81 (-11.8, 6.87)	0.47
MCOP	-2.17 (-12.4, 8.09)	0.67	3.93 (-5.77, 13.6)	0.42	-5.15 (-15.7, 7.06)	0.39
MCNP	-2.64 (-11.7, 6.45)	0.57	2.70 (-6.31, 11.7)	0.55	-3.99 (-13.9, 6.75)	0.45
ΣDEHP	2.87 (-6.17, 11.9)	0.53	1.14 (-8.61, 10.9)	0.82	5.85 (-4.81, 17.6)	0.29

CI, confidence interval, SHBG, sex hormone-binding globulin. Linear regression models adjusted for age at enrollment, prepregnancy body mass index, and urinary specific gravity.

*P < 0.05.

†P < 0.10.

## Discussion

To our knowledge the present study is the first to examine the relationships between urinary phthalate metabolites and serum thyroid hormone levels longitudinally across pregnancy. Few associations were observed in longitudinal analyses, although we did find statistically significant inverse associations between FT3 and MCPP, a non-specific metabolite of several high molecular weight phthalate plasticizers, and also between progesterone and MEP, the main metabolite of diethyl phthalate commonly used in personal care products. We also found that study visit of sample collection significantly modified the relationships between certain urinary phthalate metabolites and thyroid hormone levels. Our cross-sectional analyses corroborated our findings from the interaction analyses, and showed that associations between certain urinary phthalate metabolites and FT3 and FT4 hormone levels differed markedly (in terms of both magnitude and significance) based on visit of sample collection. For visit 1, associations with FT3 and FT4 were generally inverse, but at visit 3 these inverse relationships were stronger. Taken together, these results suggest that phthalate exposure during pregnancy may alter maternal thyroid and sex hormone levels. They also suggest that the magnitude of the potential endocrine-disrupting effect of phthalates may depend on the timing of exposure during gestation.

Of the thyroid hormones, we observed that FT4 was inversely associated with certain urinary phthalate metabolites at visit 3 (26 ± 2 weeks of gestation). These results are in contrast to a previous cross-sectional investigation conducted among 76 Taiwanese pregnant women with urinary phthalate metabolites and hormone levels measured in the second trimester (mean time of sample collection = 27.9 ± 2.3 weeks of gestation) [23]. Huang and colleagues reported a statistically significant inverse association between urinary MBP and serum FT4 after adjusting for similar covariates in their regression model. We did not observe an analogous association at visit 3 (a comparable gestational age at sample collection) in the present study. FT3 was not measured in this previous investigation and regression results for TSH were not presented by the authors, thereby precluding additional comparisons with findings from our analyses. Differences between the results of our study and the Taiwanese study may be due to dissimilarities in study design, participant demographic characteristics, exclusion/inclusion criteria and/or exposures. For example, the median concentration (unadjusted for urinary dilution) of MBP was approximately four times higher in the Taiwanese pregnant women (81.8 ng/mL) [23] compared to the corresponding concentration found in women in the PROTECT cohort (20.1 ng/mL) [31].

Our findings for urinary metabolites of DEHP and serum FT4 and TSH are consistent with a previous study of adults (including non-pregnant women and men) participating in NHANES that reported significant inverse associations between certain urinary DEHP metabolites and FT4, although that larger study also reported positive relationships between these metabolites and TSH which were not significant in the present study [15]. Our results for FT3 and FT4 are also in agreement with the limited animal studies in which rats fed DEHP-contaminated diets, at doses orders of magnitude higher than those experienced by the PROTECT participants, had lower plasma T4 levels compared to controls and plasma T3 levels remained unchanged [42–45].

Various biological mechanisms have been proposed through which environmental chemicals may exert their action on thyroid function. Thyroid-disrupting chemicals may target the hypothalamic-pituitary-thyroid axis at multiple levels and may disrupt thyroid hormone homeostasis by interfering with the synthesis and regulation by the hypothalamic-pituitary thyroid hormones (i.e., thyrotropin-releasing hormone [TRH] and TSH), binding of thyroid hormones to distributor proteins, cellular uptake mechanisms of thyroid hormones, metabolism of thyroid hormones by iodothyronine deiodinases, transcriptional activity of thyroid hormone receptors and/or receptor activation [46, 47]. It has been suggested that phthalates may bind to thyroid hormone receptors, consequently activating or inhibiting thyroid hormone action [48–50], although data overtly demonstrating the binding of phthalates to thyroid receptors are lacking. Available experimental studies have provided some evidence for these potential mechanisms of thyroid disruption. *In vitro* studies have shown that phthalates may alter the sodium/iodide symporter-mediated uptake of iodide by thyroid follicular cells [51, 52], exhibit thyroid receptor antagonist activities [24, 53, 54], or displace thyroid hormones (e.g., T3) from distributor proteins [55]. Additionally, phthalates were found to alter the transcription of genes involved in the hypothalamic-pituitary-thyroid axis as well as the whole-body content of thyroid hormones in zebrafish [56].

Of the sex hormones, we observed a consistent inverse relationship between urinary MEP and serum progesterone across the two time points in pregnancy. In contrast to these findings, the previously described study by Huang et al. [23] reported no relationship between urinary MEP or other phthalate metabolites and progesterone during approximately the same sampling period in pregnancy as visit 3 in the current study. At visit 1 (18 ± 2 weeks of gestation), we observed a marginally significant inverse relationship between urinary MEP and SHBG, although no association was observed at visit 3. These results are in agreement with a previous study conducted among 1,377 pregnant Australian women, in which an inverse relationship between serum MEP and SHBG concentrations was observed early in pregnancy (18 weeks of gestation) [30]. Animal studies investigating the potential toxicity of phthalates on the female reproductive system are scarce, and have primarily examined effects of DEHP and DBP. These phthalates have been shown to reduce progesterone production in female rats *in vivo* and in rat granulosa cells *in vitro* [57–59]. However, we did not observe these relationships with metabolites of DEHP and DBP in the present analysis.

Our null findings for estradiol were comparable to those of previous epidemiologic studies conducted among pregnant women [23, 29]. However, available toxicological data have shown that the ovary, in particular the granulosa cells of preovulatory follicles, may be the primary target site for DEHP [60]. Specifically, *in vivo* and *in vitro* studies of DEHP-treated female cycling rats have demonstrated that suppressed estradiol production may be the principal functional modification by DEHP [60–62]. These toxicology studies suggest that phthalates have the potential to induce alterations in steroidogenesis in female animals, although further research is needed to fully characterize the specific modes of action. Examining this association in a larger sample of pregnant women may enable identification of subtler relationships.

While the timing of exposure to endocrine disrupting chemicals during pregnancy has been shown to influence both the severity and onset of adverse developmental and reproductive outcomes [63], we are aware of no epidemiologic studies that have attempted to identify periods of susceptibility to phthalate-induced alterations in thyroid and sex steroid hormone levels in pregnant women. During the first trimester, the fetus relies solely on maternal T3 and T4 until the fetal thyroid gland fully develops at approximately 10 weeks of gestation [64]. Thus, maintaining maternal euthyroidism during the first trimester is critical – even slight alterations in maternal thyroid hormones during this period of gestation has been associated with deleterious neurodevelopmental and reproductive outcomes [65–67]. In later pregnancy, maternal thyroid hormones are essential for fetal thyroid homeostasis [68]. Although we detected a significant inverse relationship between urinary MiBP and serum FT4 in the first trimester (i.e., at visit 1), the strongest findings for FT4 were observed in the second half of pregnancy (i.e., at visit 3). Despite epidemiological investigations that have shown the potential adverse reproductive consequences of overt thyroid disease in early and late gestation [69, 70], the impact of trimester-specific subclinical alterations in maternal thyroid function on pregnancy outcomes remains largely understudied. Furthermore, we observed a consistent inverse association between urinary MEP and progesterone at each study visit, and this effect was greatest earlier in pregnancy (i.e., visit 1). Because progesterone plays an essential physiological role in the establishment and maintenance of pregnancy, insufficient concentrations of this hormone throughout gestation may lead to pregnancy loss or preterm birth, depending on the timing of hormonal disruption [12, 71, 72].

While our findings may have important public health implications, our study has several limitations. Characteristic of most preliminary analyses in an ongoing prospective cohort, our study was limited by a small sample size. We expected that the availability of multiple measurements per subject would increase the power to detect associations, but analysis revealed that the effects of phthalates on hormone disruption may be different depending on timing of exposure. Furthermore, given the large number of statistical tests performed in the present analyses, there is possibility of type-1 error. Another potential limitation is our timing of data collection during mid-pregnancy, which may potentially bias our results since women who spontaneously aborted in the first trimester are not captured in our analyses. We do not have biological data from these women pertaining to the exposure and outcomes of interest to assess the direction of the potential bias. Our study may also have been limited by our evaluation of circulating thyroid hormones as a sole indicator of thyroid toxicity [73]. Measurements of peripheral thyroid hormones may not fully capture the phthalate-induced effects on thyroid homeostasis [68]. That is, blood levels of thyroid hormones may not correspond with actions at the receptor, such as regulation of gene expression and the developmental processes in which they are involved [73, 74]. However, given the limited amount of data on this subject and the infeasibility of collecting more specific markers during pregnancy, levels of circulating thyroid hormone measurements may serve as the most appropriate biomarker of thyroid disruption in pregnant women. Additional studies are necessary to address potential phthalate-induced alterations in thyroid-hormone responsive genes relevant to pregnancy outcomes. It may also be possible that the serum measurements included in our analyses represented transient changes in thyroid and sex hormone levels that may not have persisted outside of the two measurement time points. However, even temporary and/or subclinical alterations in maternal hormone levels may be biologically relevant and may induce permanent effects on parturition and/or the developing fetus [68]. Our study was also limited by the lack of information concerning the iodine and selenium status of our study participants, which may be important because deficiencies in these trace elements can impair normal thyroid hormone function [75]. However, we have no reason to expect that these would be associated with phthalate exposure, so any deficiencies in these substances would affect the precision of the effect estimates rather than the effect estimates themselves [76]. Finally, our study was conducted among pregnant women in Northern Puerto Rico, which may have implications for the generalizability of results.

Despite these limitations, our study had several strengths. The collection of biomarker measurements at two time points during pregnancy enabled us to utilize mixed modeling techniques to more powerfully detect associations among repeated measurements and also evaluate potential periods of gestation during which phthalates may have a more profound impact on maternal hormone levels. Also, as pregnancy is characterized by a dynamic interplay between maternal endocrine hormones, our measurements of hormone concentrations from two disparate endocrine axes is advantageous. Each set of hormones may represent distinct mechanisms through which phthalates may influence downstream reproductive health outcomes. Furthermore, we used novel and precise analytical techniques to measure serum levels of FT3 and FT4. Especially pertinent to a population of pregnant women, this method has advantages over traditional immunoassays because it is more specific, does not cross-react with other analytes, and is not influenced by serum binding proteins [35, 77, 78]. Notably, thyroxine binding proteins may increase as much as 50 percent during pregnancy [78].

## Conclusions

The results of our study provide suggestive evidence for phthalate-induced endocrinal disturbances during pregnancy, and may augment mechanistic understanding of the impact of phthalates on reproductive health outcomes. Future research on the specific pathways through which phthalates may alter thyroid and sex hormone concentrations are required for targeted interventions aimed at preventing downstream hormone-mediated adverse reproductive health outcomes in pregnant women.

## Electronic supplementary material

Additional file 1: Table S1: Urinary phthalate metabolite concentrations (ng/mL) in pregnant women from Puerto Rico (N = 106). (DOC 62 KB)

Additional file 2: Table S2: Pearson correlations between serum sex hormones, serum thyroid hormones, and maternal age in pregnant women from Puerto Rico (N = 106). (DOC 32 KB)
