# Supplementary material for: Urinary phthalate metabolites in relation to maternal serum thyroid and sex hormone levels during pregnancy: a longitudinal analysis
Source: Reprod Biol Endocrinol. 2015 Jan 17;13:4. doi: 10.1186/1477-7827-13-4 (PMC4326411; doi:10.1186/1477-7827-13-4)
Supplement: Supplementary file 1 — Additional file 1: Table S1: Urinary phthalate metabolite concentrations (ng/mL) in pregnant women from Puerto Rico (N = 106). (DOC 62 KB) [file 12958_2014_1304_MOESM1_ESM.doc]

| **Supplemental Table S1 – Urinary phthalate metabolite concentrations (ng/mL) in pregnant women from Puerto Rico (N=106)†** | | | | | | | | |
| --- | --- | --- | --- | --- | --- | --- | --- | --- |
|  |  |  |  | **Percentiles** | | | | |
|  | **GM (GSD)** | **p-value*** |  | **25th** | **50th** | **75th** | **95th** | **Max** |
| MEHP |  |  |  |  |  |  |  |  |
| Visit 1 | 3.22 (2.90) | 0.98 |  | 1.61 | 3.14 | 6.36 | 13.5 | 50.9 |
| Visit 3 | 3.24 (2.79) |  |  | 1.69 | 3.08 | 6.73 | 19.7 | 32.8 |
| MEHHP |  |  |  |  |  |  |  |  |
| Visit 1 | 10.7 (2.61) | 0.98 |  | 6.14 | 10.5 | 19.9 | 37.9 | 290 |
| Visit 3 | 10.8 (2.27) |  |  | 7.28 | 11.1 | 16.9 | 42.0 | 88.2 |
| MEOHP |  |  |  |  |  |  |  |  |
| Visit 1 | 9.09 (2.49) | 0.95 |  | 5.57 | 8.33 | 16.5 | 29.0 | 259 |
| Visit 3 | 9.41 (2.26) |  |  | 6.22 | 9.86 | 14.8 | 38.6 | 64.7 |
| MECPP |  |  |  |  |  |  |  |  |
| Visit 1 | 20.4 (2.27) | 0.97 |  | 12.7 | 20.8 | 31.4 | 61.0 | 712 |
| Visit 3 | 20.2 (1.96) |  |  | 13.4 | 20.8 | 29.3 | 69.7 | 121 |
| MBzP |  |  |  |  |  |  |  |  |
| Visit 1 | 3.62 (3.16) | 0.54 |  | 1.74 | 3.73 | 7.65 | 27.0 | 59.0 |
| Visit 3 | 3.60 (3.19) |  |  | 1.49 | 3.22 | 6.99 | 29.8 | 108 |
| MBP |  |  |  |  |  |  |  |  |
| Visit 1 | 19.1 (2.99) | 0.81 |  | 10.8 | 19.3 | 35.3 | 112 | 278 |
| Visit 3 | 19.1 (2.48) |  |  | 10.4 | 18.6 | 36 | 86.6 | 182 |
| MiBP |  |  |  |  |  |  |  |  |
| Visit 1 | 10.1 (2.47) | 0.46 |  | 5.97 | 10.3 | 17.9 | 35.2 | 157 |
| Visit 3 | 11.5 (2.61) |  |  | 6.33 | 10.9 | 16.9 | 61.6 | 654 |
| MEP |  |  |  |  |  |  |  |  |
| Visit 1 | 96.9 (5.39) | 0.95 |  | 24.2 | 97.7 | 365 | 1860 | 6910 |
| Visit 3 | 106.1 (6.36) |  |  | 24.7 | 83.6 | 354 | 2410 | 7640 |
| MCPP |  |  |  |  |  |  |  |  |
| Visit 1 | 1.98 (2.52) | 0.09 |  | 1.04 | 2.08 | 3.02 | 8.00 | 82.1 |
| Visit 3 | 2.27 (2.70) |  |  | 1.24 | 1.86 | 3.52 | 14.8 | 64.8 |
| MCOP |  |  |  |  |  |  |  |  |
| Visit 1 | 14.9 (2.93) | 0.02 |  | 7.96 | 13.4 | 24.8 | 89.8 | 1060 |
| Visit 3 | 18.5 (3.32) |  |  | 8.23 | 15.8 | 36.0 | 214 | 939 |
| MCNP |  |  |  |  |  |  |  |  |
| Visit 1 | 2.42 (2.56) | 0.13 |  | 1.33 | 2.12 | 3.73 | 14.9 | 51.6 |
| Visit 3 | 2.24 (2.24) |  |  | 1.34 | 2.06 | 3.23 | 11.3 | 33.6 |
| GM, geometric mean, GSD, geometric standard deviation.  †All concentrations adjusted for specific gravity. *Test for fixed effects from linear mixed models with random intercepts. | | | | | | | | |
|  |  |  |  |  |  |  |  |  |
