# Supplementary material for: Urinary phthalate metabolites in relation to maternal serum thyroid and sex hormone levels during pregnancy: a longitudinal analysis
Source: Reprod Biol Endocrinol. 2015 Jan 17;13:4. doi: 10.1186/1477-7827-13-4 (PMC4326411; doi:10.1186/1477-7827-13-4)
Supplement: Supplementary file 2 — Additional file 2: Table S2: Pearson correlations between serum sex hormones, serum thyroid hormones, and maternal age in pregnant women from Puerto Rico (N = 106). (DOC 32 KB) [file 12958_2014_1304_MOESM2_ESM.doc]

| **Supplemental Table S2 – Pearson correlations between serum sex hormones, serum thyroid hormones, and maternal age in pregnant women from Puerto Rico (N=106)** | | | | | | | |
| --- | --- | --- | --- | --- | --- | --- | --- |
|  | Progesterone † | Estradiol | SHBG | TSH † | Free T3 | Free T4 | Age |
| Progesterone † | 1.00 | 0.66* | 0.23* | 0.05 | -0.14* | -0.17* | -0.12 |
| Estradiol |  | 1.00 | 0.29* | 0.10 | -0.08 | -0.27* | -0.13 |
| SHBG |  |  | 1.00 | 0.06 | -0.10 | -0.11 | -0.09 |
| TSH † |  |  |  | 1.00 | 0.06 | -0.01 | -0.17* |
| Free T3 |  |  |  |  | 1.00 | 0.17* | -0.11 |
| Free T4 |  |  |  |  |  | 1.00 | -0.10 |
| Age |  |  |  |  |  |  | 1.00 |
| TSH, thyroid-stimulating hormone, SHBG, sex hormone-binding globulin.  † Log-transformed in statistical analysis.  *P<0.05 | | | | | | | |
